# Single Camera Face Position-Invariant Driver’s Gaze Zone Classifier Based on Frame-Sequence Recognition Using 3D Convolutional Neural Networks

**DOI:** 10.3390/s22155857

**Published:** 2022-08-05

**Authors:** Catherine Lollett, Mitsuhiro Kamezaki, Shigeki Sugano

**Affiliations:** 1 Graduate School of Creative Science and Engineering, Waseda University, Tokyo 169-8555, Japan; 2 Research Institute for Science and Engineering (RISE), Waseda University, Tokyo 162-0044, Japan

**Keywords:** driver monitoring, gaze classification, convolutional neural networks

## Abstract

Estimating the driver’s gaze in a natural real-world setting can be problematic for different challenging scenario conditions. For example, faces will undergo facial occlusions, illumination, or various face positions while driving. In this effort, we aim to reduce misclassifications in driving situations when the driver has different face distances regarding the camera. Three-dimensional Convolutional Neural Networks (CNN) models can make a spatio-temporal driver’s representation that extracts features encoded in multiple adjacent frames that can describe motions. This characteristic may help ease the deficiencies of a per-frame recognition system due to the lack of context information. For example, the front, navigator, right window, left window, back mirror, and speed meter are part of the known common areas to be checked by drivers. Based on this, we implement and evaluate a model that is able to detect the head direction toward these regions having various distances from the camera. In our evaluation, the 2D CNN model had a mean average recall of 74.96% across the three models, whereas the 3D CNN model had a mean average recall of 87.02%. This result show that our proposed 3D CNN-based approach outperforms a 2D CNN per-frame recognition approach in driving situations when the driver’s face has different distances from the camera.

## 1. Introduction

According to various reports, countless road accidents have happened throughout the years as a result of driver distraction. One of the primary causes of the great majority of accidents is an inattentive driver. According to annual data, about half a million people are injured and thousands die as a result of these events [1,2,3].

If our cars can offer Advanced Driving Assistance Systems (ADAS) that detect distractions in advance, the system will be able to not only alert, but also take control of the situation in our near future autonomous vehicles, minimizing large-scale traffic accidents and contributing to traffic safety.

Inside an ADAS, a driver’s gaze zone classifier is critical for recognizing the driver’s level of awareness [4,5], workload [6], and readiness [7] so that a partial or complete autonomous vehicle can take control when a dangerous situation arises. Examples of dangerous situations include when the driver is drowsy or focused on tasks other than driving. Several previous studies have implemented this kind of classifier. However, their evaluations were conducted under restricted conditions. Making this classification in an unconstrained context is still extremely difficult, resulting in very poor results across already conducted studies.

Our research line focuses on developing a high-performance driver gaze classifier under unconstrained conditions. Having a high-performance system in unrestricted conditions implies being able to identify distractions with fewer mistakes and thereby reducing automobile accidents significantly.

**Figure 1:**
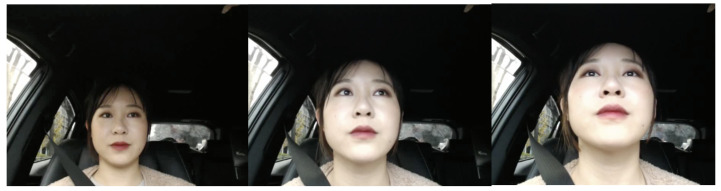
Left: Driver’s face facing front with a large distance to the camera. Middle: Driver’s face facing front with a middle distance to the camera. Right: Driver’s face facing front with a small distance to the camera.

While implementing our previous studies [8,9,10], we noticed that different distances from the camera is one unconstrained condition that may lead to misclassification. This specific condition, which can occur at any time during a driving scenario, is not clearly evaluated in any of the existing approaches’ experiments. Furthermore, the datasets used in previous research do not include data where the driver’s camera distance differs significantly. This is why we collected data, analyzed it, and suggested a model that can be robust in the event that this situation arises. Figure 1 illustrates the problem by showing different distances of the driver’s face from the camera.

The majority of the existing approaches adhere to a single-frame feature-based system. However, it seems that using spatio-temporal characteristics helps to make the classification more robust in situations where the pattern varies widely. Different works reinforce the benefits of the accuracy that comes along with having the spatio-temporal feature inside their models [11,12]. Considering this, we decided to make a model using 3D CNN, which considers spatio-temporal features, for classifying six different head positions towards different standard driving gaze areas [13,14]: head facing front, head facing right window, head facing navigator, head facing left window, head facing back mirror, and head facing speed meter.

Table 1 lists all the head positions proposed as labels for our model. Each label is composed of two letters: one for the head direction and one for the eye direction. For example, FF stands for face facing front, eyes facing front. This is based on our prior works [8,9], where it is important to make clear the differences between where the face and eyes are facing.

We compared the suggested 3D CNN model to a 2D CNN model to compare a per-frame recognition system, which is the common implementation in prior research, and a sequence frame recognition. We addressed the advantages and disadvantages of each technique, as well as why the 3D CNN model outperformed the 2D CNN model.

To summarize, the five key contributions of our work are:

1. We evaluated our model by focusing on the challenge of the driver being at different distances from the camera. To understand better how this problem might reduce accuracy while making a classification and how to overcome it, we gathered data, implemented a model, analyzed the obtained results, and wrote a discussion.
2. We propose applying a 3D convolution operation to extract spatial and temporal features from videos and overcome this problem. We evaluate and demonstrate the benefits of implementing it in driving situations where the driver has different distances from the camera.
3. Since a per-frame-based recognition is often used inside gaze classifiers, we evaluate the proposed 3D CNN baseline model against a 2D CNN baseline model. The experimental results show that the proposed 3D model can outperform the 2D CNN model overall.
4. We propose a model portable and extensible system since we used only one camera and no external sensors for acquiring the data.
5. We propose a model that can correctly classify new distinct subjects.

The rest of this paper is organized as follows: Related works are discussed in Section 2. Section 3 describes the methodology used for this work and the details of our model. The experimental setting and results are reported in Section 4. Section 5 summarizes this work’s contribution to intelligent vehicles, and finally, Section 6 concludes and discusses future works.

## 2. Related Works

Keeping track of the driver’s gaze direction may aid in identifying the driver’s attention and avoiding automobile accidents when driving on a daily basis. Unfortunately, these driving scenarios are frequently exposed to a series of challenging conditions. In this paper, we are especially interested in proposing a methodology that can ease the misclassification due to different distances of the driver’s face from the camera.

To the best of our knowledge, none of the existing gaze classifiers evaluates their models against a dataset of diverse driving positions from the camera. However, driver gaze classifiers have a long history. We review the existing gaze classifiers that use a single camera, or sensor, to offer readers a background on their methodology.

Ref. [15] tries to compare the performance of a driver gaze classifier using a model that uses a head and eye pose versus just using a head pose. Head pose corresponds to the head direction. For instance, if the head is facing towards the speed-meter, the head pose is towards the speed-meter. Their pipeline’s steps are: face detection and face alignment using a Histogram of Oriented Gradients (HOG) combined with a linear Support Vector Machine (SVM) classifier, pupil detection, feature extraction and normalisation, and classification using a random forest classifier.

Ref. [16] also uses Haar feature-based methodologies to make their classification. First, they make a coarse head pose detection with different frontal, left and right-profile face detectors. Then, they extract the gaze feature descriptor only for the frames that have frontal poses. Next, they detect the left iris, right iris, mouth and nose areas. All the detections are made based on fast AdaBoost cascade classifiers operating on Haar features descriptors. The extracted feature vector is input to a multi-class linear SVM classifier that outputs an estimated gaze direction out of eight possible directions. Then, a temporal post-filtering is used to produce the final prediction of gaze in the current frame, which consists of a sliding window history of class labels from the past five frames.

Ref. [17] considers a pupil center corneal reflection (PCCR)-based technique. They begin by acquiring the driver’s frontal face and then utilize the dlib facial feature tracker, to determine facial landmarks using a near-infrared camera frame as input. After this, they use the associated landmarks to obtain images of the face, left eye, and right eye. Then, using three distinct CNNs, they construct three sets of feature values, calculate their Euclidean distances, and classify the gaze zone based on the score fusion of these three distances.

As it occurs in these works, face and facial landmark identification is used in a substantial majority of gaze classifiers as a very initial stage. This stage is critical since it retrieves the basic data for the remaining process. If this step fails, the rest of the classification will fail. For this step, refs. [15,16] rely on Cascade Classifiers operating on Haar features descriptors, HOG, and/or SVM. According to [18], when striving to detect facial landmarks under unconstrained settings, these algorithms incorrectly locate the faces or generate a misaligned estimate of the landmarks. Despite having the poorest recognition, it most likely has the highest computing speed.

Ref. [19] includes face detection, landmark estimation, head pose, and eye cues inside their pipeline. For face detection, they use Viola-Jones’s AdaBoost cascade with Haar features that is similar to the implementation of Faceness. For landmark detection, they use estimation from cascaded regression models as described in [20].

To address the absence of an annotated dataset for gaze zone estimation, ref. [21] employed a transfer-learning approach with a pre-trained CNN model to project the gaze estimation problem via regression on mobile devices with big and trustworthy datasets into a new classification task. They divided the car’s zone into ten zones and collected the data with different iOS devices, such as iPads and iPhones. They altered the iTracker’s deep CNN by replacing the SVM with the last fully connected (FC) layer that links to the Euclidian regression. We think one strong point of [21] is that it can run in low-capacity devices.

Ref. [22] based their algorithm on detecting head pose and landmark detection. They based the head pose algorithm on Posing from Orthography and Scaling with Iterations (POSIT), which assesses the 3D posture of an object in a camera frame. This technique primarily solves object poses by utilizing the relationship between non-coplanar space locations and their two-dimensional image. The procedure from coarse to fine is used to locate the pupil and the corner of the eye. The exact placements of the eyes and the corner of the eye are then determined using Local Binary Features (LBF) shape regression. Then they use random forest to make the classification.

Ref. [23] fine-tuned four different popular CNN architectures, AlexNet, VGGNet, ResNet, and SqueezeNet, to compare the results of using the different pre-train CNN models.

For us, ref. [24]’s approach is very interesting. The driver’s perception of external stimuli is examined by observing the motion of the gaze and the environment. This approach is described as vision-in/vision-out. However, a head-eye-tracker provides gaze direction measurements, which can be intrusive to the drivers in an actual driving situation. Nonetheless, we think their approach is exciting, and mapping the objects with the driver’s gaze will help reach a higher accuracy inside gaze classifiers. Ref. [13], in the same line, uses a head-eye-tracker that is intrusive for the driver. Ref. [25] instead uses a standalone eye tracker. However, as they explain, the eye tracker was not always able to correctly capture the gaze because of the constantly changing light conditions, which produced disturbing reflections on the device [25].

Ref. [26], for face detection, used the Viola-Jones face detector and incorporates a multi-zone Iterative Closest Point (ICP)-based head location tracking and gaze estimation based on appearance using an RGB-D camera. In the experiments, the driver’s face always appears entirely in the field of view.

Ref. [27] presents a random forest-trained classifier with head vectors and eye image features. In this study, POSIT computes the head vector when a 3D face model is combined with facial landmark recognition. They employ the Supervised Descent Method (SDM) facial landmark detector to identify the eye corner points around the eyeball and other face points while recognizing facial landmarks.

Refs. [28,29,30] only use the face’s pose information for their classification. Ref. [28] formulates intervals based on continuous gaze angles and treats quantized angles’ grid as an image for dense prediction using a headband. In our opinion, the usage of a band is intrusive to the driver. Ref. [29] introduces a system based on existing CNN structures with slight modifications; the pose is estimated with POSIT algorithm with a 3D generic face and selected rigid landmarks. Ref. [30]’s approach uses a point cloud that can be robust to large head poses, but their method uses Fi-Cap, which can be an intrusive device.

Ref. [31]’s approach is very interesting for us because it tries to overcome the eyeglass reflection challenge that constantly occurs in a driving scenario.

Ref. [32] uses a Fine-Grained Head Pose Estimation Without Keypoints. The basic idea of this method is to use the Euler angles for detecting the head pose from image intensities combining binned pose classification and regression [33]. Ref. [32]’s pipeline includes face detection, the estimation of head pose angles, estimation of facial and eye landmarks, calculation of confidence values, and fully connected network architecture.

Refs. [8,9,10] is our research line to improve the robustness of gaze zone classifiers. In [8], we implemented a system that can classify a driver’s gaze robustly when the face and eyes are in different directions without external occlusions such as masks, scarves, or eyeglass reflection. Ref. [10] was our first step in creating a classifier that can be robust to external occlusions, such as masks or scarves. We are still working on the problem that comes with eyeglass reflections.

While implementing our previous work, we realized that the distance from the camera could be a high factor in classification, so we aimed to analyze and propose an enhanced approach that makes another step toward a robust classification in scenarios where unconstrained conditions are exposed.

The spatio-temporal correlation feature that comes with having context makes 3D CNN superior to 2D CNN. Different works, unrelated to driver’s gaze zone classification, show the advantages of using a 3D CNN over a 2D CNN. Ref. [11] considers the automated recognition of human actions in surveillance videos. Understanding the limitations of 2D inputs, they compared a 3D CNN model and a 2D CNN model. Their results show that the 3D CNN model outperforms the other methods, demonstrating a better performance in real-world setting environments. As a reference, ref. [12] proposes to extend their work using a 3D FCN (Fully Convolutional Neural Network) model to further enhance the performance of object detection in a point cloud. First, they made a 2D FCN that achieved notable performance in image-based detection tasks, and then they extended it to a 3D model. In their work, using an end-to-end approach, they detect objects and estimate oriented object bounding boxes. Their object detection approach can be generalized to other tasks on point cloud captured by Kinect, stereo, or a monocular structure from motion.

To our best knowledge, all the previous work related to driver gaze classification is per-frame system-based, and none of them takes advantage of the features that video recognition comes with. However, there are a limited number of studies that analyze drivers’ drowsiness state while using 3D CNNs. In [34], the authors propose driver drowsiness detection based on a condition-adaptive representation learning framework based on a 3D CNN model. Their experimental results show that their 3D CNN-based framework outperforms the existing drowsiness detection methods based on visual analysis. In the same line for detecting a driver’s drowsiness, ref. [35] applies a 3D CNN to extract features in the spatial-temporal domain; then they use gradient boosting for a drowsiness classification, and finally, they propose semi-supervised learning to enhance the performance overall. Still, it is unclear how robust these classifiers can be toward the misclassification concerning the camera’s different positions.

Therefore, our research aims to reach a classifier with high performance during real driving scenarios where the driver’s face is in different positions in regard to the camera. This is an approach for implementing a more robust driver’s gaze classifier in a natural real-world setting.

## 3. Methodology

The purpose of this research is to evaluate and contrast the performance of a per-frame recognition system (2D CNN) against a sequence-frame recognition system (3D CNN) to make a gaze zone classifier. In this study, we analyze a dataset in which the driver is at various distances from the camera. We aim to demonstrate how the context may assist in reducing misclassifications in the above-discussed scenario.

In this paper, we demonstrated that using spatio-temporal features is possible to improve misclassifications during:

- Frames caused by blurred images while using a per-frame recognition system.
- Situations where a driver has different distances from the camera.

After this, we compare the performance of the 3D CNN model and the 2D CNN model. The 2D CNN model is often used as a base in already existing gaze classifier models.

Convolutions are learnable filters or matrices used in neural networks to extract low-dimensional properties from input data. They can preserve spatial or positional relationships between input data components. Convolutional neural networks make use of spatially local correlation by building a local connection pattern between neurons in subsequent layers. Convolution is easily characterized as the step of applying the sliding window technique to the input and producing a weighted sum as the output. The weighted sum of the feature space is used as the input for the subsequent layers.

On image datasets, CNN designs often use 2D convolutional filters. The fundamental premise of 2D convolutions is that the convolutional filter operates in two directions (x, y) to generate low dimension characteristics from an image input. The resulting form is a two-dimensional matrix as well.

Three-dimensional convolutions, on the other hand, use a three-dimensional filter that operates in three dimensions (x, y, and z) to construct low-level feature representations. Their final shape is a three-dimensional volume space, similar to a cube. They may be used to detect events in videos or 3D medical images.

### 3.1. Face Detector

Both models use facial detection as an input. Unconstrained driving conditions may result in a high number of face detection misclassifications; therefore, selecting a robust face detector is critical. Different previous works rely on Cascade Classifiers operating on Haar Features Descriptors [36] or HOG [37] and multi-class linear SVM for face detection. However, their proposal could not be robust enough in different situations, e.g., profile faces. In comparison to its HOG detector, Dlib has built a fast-to-use library with a proprietary architecture for a CNN-based detector that is capable of identifying faces practically at all angles. We detect the face using Dlib CNN Face Detector since it is a fast-to-use package with high performance on the dataset used in this work.

However, as we highlighted in our previous work, it is important to select even more robust face detectors to avoid misclassifications when partial occlusions are present, such as situations while the driver is wearing masks or scarves or there are eyeglass reflections. We recommend reading [38] lists that provide the most robust face detection classifiers as a short reference for the reader. Based on prior experience, Single Shot Scale-invariant Face Detector (S3FD) has a very strong performance and outperforms other benchmark face detectors by a large margin across the different face detection benchmark datasets as Annotated Faces in the Wild (AFW), PASCAL face, Face Detection Dataset and Benchmark (FDDB), and WIDER FACE running at 36 FPS on a Nvidia Titan X for VGA-resolution images [38,39].

### 3.2. Two-Dimensional CNN Model

We built our architecture with realistic driving circumstances, precision, and speed in mind.

In this specific study, the key reason we chose CNN’s architecture over other alternatives is its powerful feature of equivariance:

(1) $$T_{\Delta x}\left(C_{k}\left(f\right)\right)=C_{T_{\Delta x}k}\left(f\right)=\left(C_{k}\left(T_{\Delta x}\left(f\right)\right)\right)$$

where $C_{k(f)}$ is the convolution operator that acts on an *f* signal of a Kernel *k* and $T_{\Delta x}$ is the translation operator vector.

With this equation, the CNN comes with the characteristic of being spatially invariant. Invariance implies that a pattern can be recognized even if its appearance varies. In a realistic driving situation, we need to keep track of the salient pattern inside the image, since we want to recognize that pattern even with variations on it. In our case, the pattern of a driver facing the back mirror will vary depending on how close the person is to the camera. This is why this particular characteristic is so valuable.

**Figure 2:**
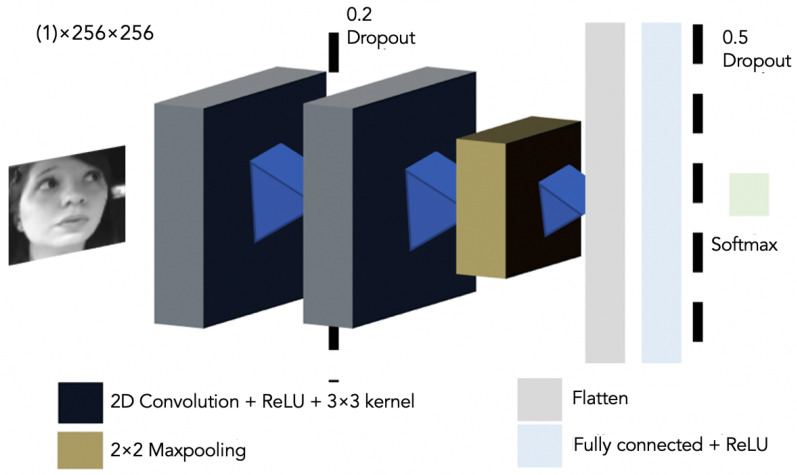
Two-dimensional CNN Network Architecture used to predict the six different proposed standard driving gaze areas with a one-channel (1) image input.

The network architecture of the 2D CNN is shown in Figure 2. Its details are explained as follows:

Input: We detect the driver’s face using Dlib CNN.

The detected face image is converted into a one-channel image of 256×256 pixels. This will be the input of our network—data augmentation strategies were not implemented (translation, rotation, and flipping).

The network architecture consists of the application of 2D convolutions, each followed by a rectified linear unit (ReLU)

Network Topography:

- 1st 2D convolutional layer, 3×3 kernel with an activation function ReLU.
- Drop-out of 20% for preventing over-fitting [40].
- 2nd 2D convolutional layer 3×3 kernel with an activation function ReLU.
- Max-pooling sub-sampling layer for removing spatial information by local dimensionality [41]. After the second convolution, we use a pooling layer to reduce the size of the next layer of neurons and prevail salient features.
- Flatten layer.
- Dense layer with a maximum norm weight constraint to reduce the probability of over-fitting.

Output: Probability prediction of the proposed labels shown in Table 1. This is made by a linear operation followed by a softmax activation.

The model was not fine-tuned for two reasons:

- The domain of this research is very narrow; therefore, there is no need to use complex pre-trained networks.
- Fine-tuning a pre-trained network on a small dataset might lead to overfitting.

Learning Conditions:

- Optimisation method: Stochastic gradient descent (SGD).
- Batch size: 32.
- Number of epochs: 200.
- Loss function: Categorical Crossentropy.
- Learning rate: 0.01.

The number of layers recommended in this neural network is proportional to the accuracy gained in a controlled setting when testing the driver’s gaze zone classifier. As a result, we steadily lowered the number of layers until we found the simplest configuration that did not impact accuracy.

### 3.3. 3D CNN Model

The primary concept behind this approach is to recognize videos rather than frames. Convolutional Neural Networks have been shown to produce outstanding performance in action recognition [42]. One benefit of a 3D CNN over a 2D CNN is the ability to capture motion information by using convolution in both time and space. The spatial and temporal features are included since this approach employs both 3D convolution and 3D max pooling. As shown in [43], temporal pooling is significant for the recognition task since it better captures the spatio-temporal information of video and lowers background noise.

### 3.4. Network Architecture

**Figure 3:**
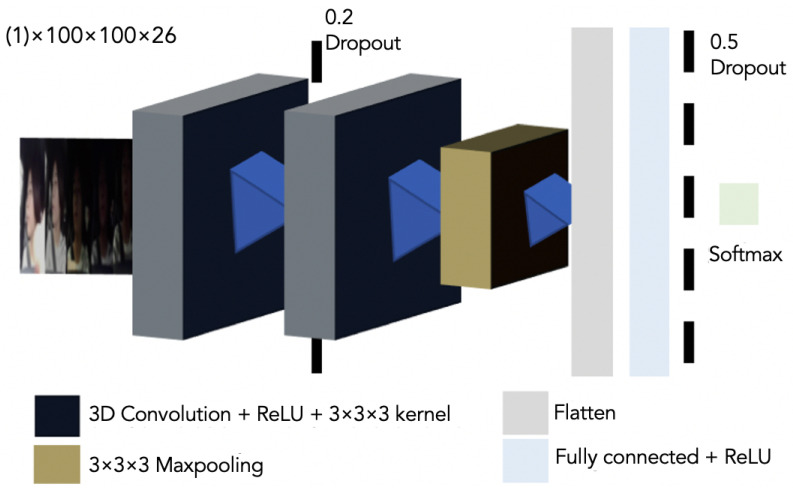
Three-dimensional CNN architecture used to predict the six different proposed standard driving gaze areas with a one-channel (1) sequence of frames input.

The network architecture is shown in Figure 3. The details are as follows:

Input: The input consists of the odd frames of a 26-frame sequence generated by the Dlib-CNN face detector. Since 3D CNN consumes memory far faster than 2D CNN, we converted the videos to a 100×100 pixels frame sequence.

Common data augmentation strategies were not implemented (translation, rotation, and flipping).

Network Topography: Since the 2D CNN model did not correctly classify in edge condition driving scenarios, we decided to adopt a similar structure to the 2D CNN model for the 3D CNN model. Hence, in this proposal, the network architecture consists of:

1. 1st 3D Convolution Layer with ReLU activation and 3×3×3 kernel.
2. Dropout of 20%.
3. 2nd 3D Convolution Layer with ReLU activation and 3×3×3 kernel.
4. A 3D Maxpooling.
5. A flatten layer.
6. A fully conected layer with ReLU activation.

Output: The last layer is also a linear operation followed by a softmax activation.

Learning Conditions: As we aim to keep the difference between the 2D CNN architecture and the 3D CNN architecture as narrow as possible, the learning conditions are equal.

- Optimization method: SGD.
- Batch size: 32.
- Number of epochs: 200.
- Loss function: Categorical Crossentropy.
- Learning rate: 0.01.

## 4. Experimental Evaluation and Results

In this research, we aim to demonstrate one clear point: by implementing a 2D and 3D CNN model for classifying a driver’s gaze zone, we compare the behavior of the 2D CNN model versus the 3D CNN model while the driver’s face is facing different distances away from the camera. In a real-world driving environment, this is a common occurrence.

### 4.1. Participants

Eighteen participants—6 females, 12 males—were involved in the experiments. The age range was between 19 are 59 years old, with an age mean of μ=26.65 and a standard deviation of σ=8.14.

- In the training dataset, the data of 4 females and 8 males were used.
- In the testing dataset, the data of 2 females and 4 males were used.

The subjects are distinct in the training dataset and testing dataset.

### 4.2. Dataset and Labeling

**Figure 4:**
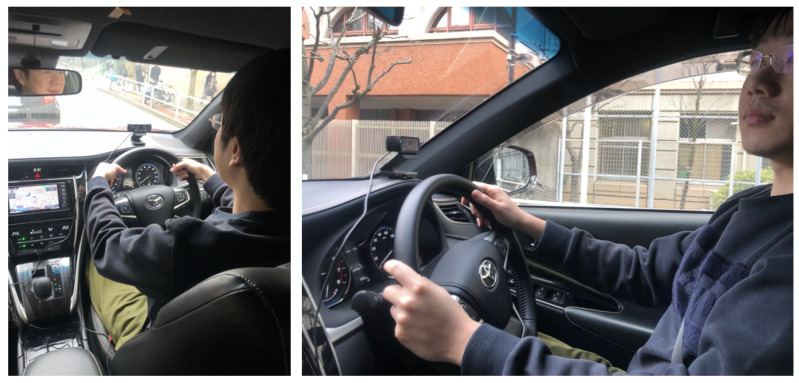
Setup of the experimental environment. The data are collected using only one camera.

We took the videos in a real car. The subjects were asked to stare at the proposed six different standard gaze zones shown in Table 1. This task should be performed three times. The difference between those three times is the face’s distance from the camera: far (70 cm ± 10 cm), middle (46 cm ± 10 cm), and near (34 cm ± 10 cm) to the camera, as shown in Figure 1. The criteria used to classify face distance (far, middle, near) was arbitrary since there is not a standardized metric to measure it. All the distances depend on the driver’s seat position. The camera is set as shown in Figure 4.

There are no external occlusions, major light variations, or occurrences where the driver stares at the backseat/back windows in the training data. However, as mentioned in the experimental setup subsection, we have instances with temporal occlusions and light variances in the testing data. The data were collected between 11 a.m. and 5 p.m. There are no videos that were taken at night-time.

**Figure 5:**
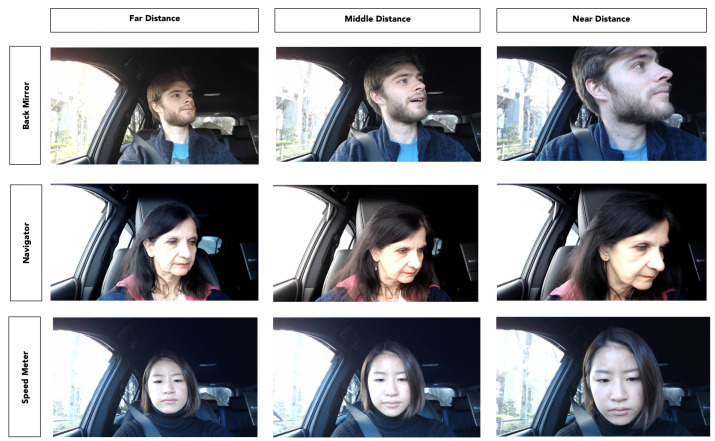
Rows from top to bottom: Driver’s face facing back-mirror, driver’s face facing navigator, driver’s face facing speed-meter. Columns from left to right: Driver’s face facing front with a far distance from the camera, driver’s face facing front with a middle distance from the camera, driver’s face facing front with a near distance from the camera.

To ensure that all situations were analyzed, data was collected in a static automobile setting for the training dataset. The frames and videos in the testing dataset, on the other hand, are a combination of real-world driving scenario settings and static car scenes. Training and testing data were collected from separate participants. Examples of the training dataset are shown in Figure 5.

### 4.3. Evaluation Metrics

For this experiment, we used Recall as the evaluation metric. Recall refers to the number of relevant instances that were fetched. The definition comes as follows:

(2) $$\mathrm{Recall}=\frac{\mathrm{True}\;\mathrm{Positive}}{\mathrm{True}\;\mathrm{Positive}+\mathrm{False}\;\mathrm{Negative}}$$

### 4.4. Experimental Setup

This section will describe how the experimental environment was created and which conditions were contained inside our dataset.

The details of the experiment go as follows:

1. For each label, there are videos with different face positions toward the camera.
2. The experiment has three kinds of condition evaluation: face with near distance from the camera, face with middle distance from the camera, and face with far distance from the camera.
3. Videos may have slight variations.
4. Videos may have occasional occlusions.
5. Eye direction is not evaluated for this experiment.
6. For 2D CNN, the classification was made for the first frame of the video.
7. For 3D CNN, the classification was made for the odd frames of a 26-frame sequence.
8. There are no videos of the same drivers in the training and test datasets.
9. To sustain our third statement in Section 1—a portable and extensible system—all the videos were captured inside a real car using a Logicool Web Camera c920r, manufacturer no. V-U0028, Tokyo, Japan (Logitech^®^, Newark, CA, USA).

### 4.5. Results and Discussion

**Figure 6:**
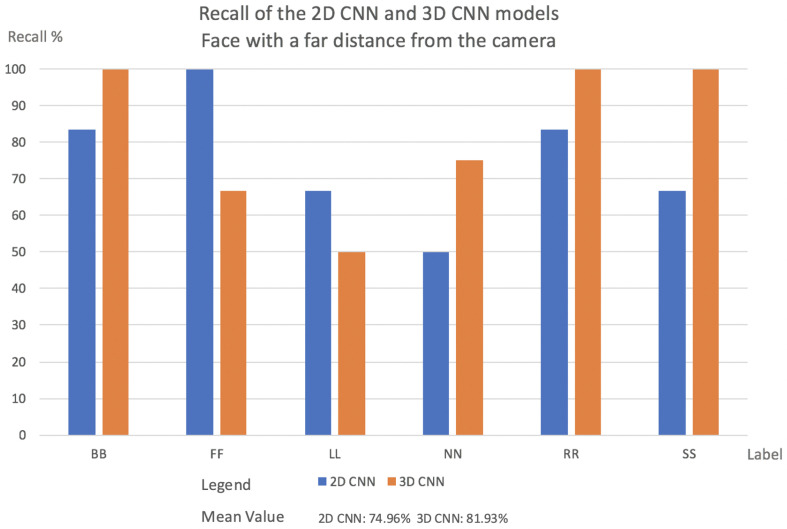
The percentage of labels successfully classified by the network in both models for videos facing the camera at a far distance.

**Figure 7:**
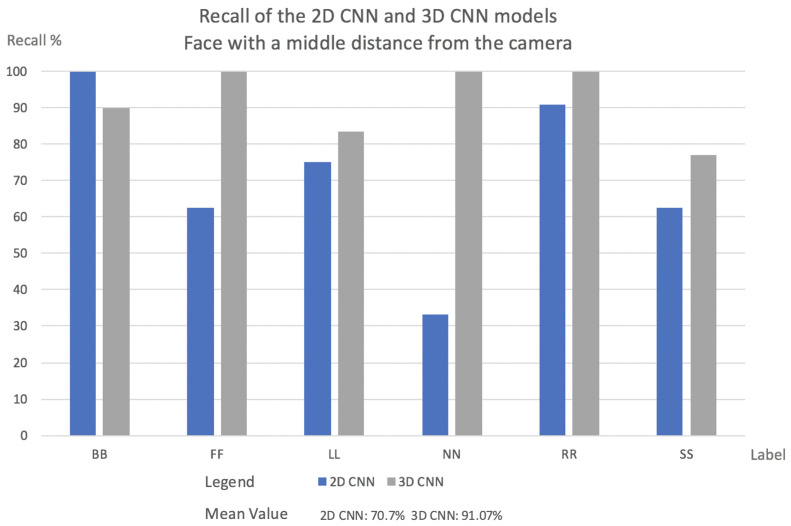
The percentage of labels successfully classified by the network in both models for videos facing the camera at a middle distance.

**Figure 8:**
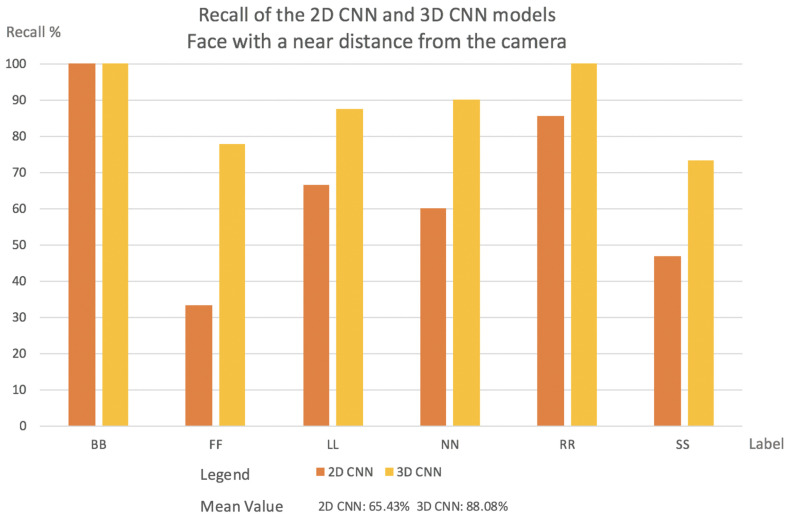
The percentage of labels successfully classified by the network in both models for videos facing the camera at a near distance.

**Figure 9:**
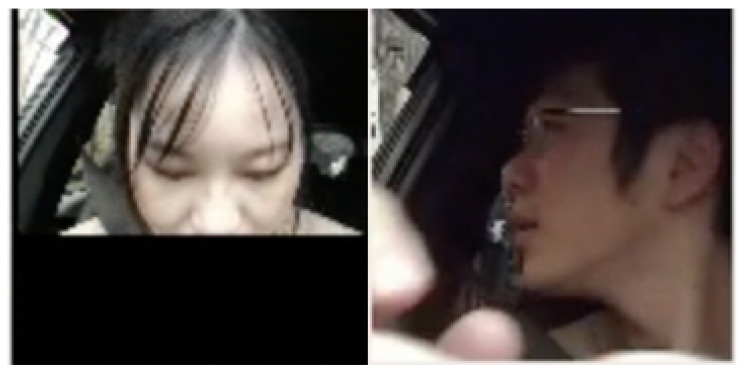
Examples of situations when misclassification may happen for 2D CNN.

This subsection will present the percentage of videos for each label that the network classified correctly in both models, 2D CNN and 3D CNN. It will also be described how each model behaves in relation to the camera’s position:

1. Videos with a far face distance from the camera results: Face with a far distance position from the camera—frequently used data in other approaches—2D CNN model and 3D CNN model has more or less same Recall. The results are shown in Figure 6.
  - In the case of FF and LL labels, the 2D CNN outperforms 3D CNN. In normal situations, when the driver is facing the front, slight movements may fall into a misclassification for the 3D CNN.
  - Two-dimensional CNN failed the classification several times when the frame was blurred.
  - Mean Value for 2D CNN model was 74.96%, while for the 3D CNN model, it was 81.93%.
2. Videos with faces positioned at a middle distance from the camera results: In this evaluation, the 3D CNN model outperformed 2D CNN in almost every case. The explanation for this is what was explained in Section 1.
  In real driving scenarios, the choice of features is highly problem-dependent since the same gaze staring situations may appear in different patterns. Instead, motion patterns seem to keep the relevant salient features independent of how near or far from the camera the driver is. The results are shown in Figure 7.
  - In the 2D CNN model, the NN label has a low recall. With the 2D CNN model, the model produces multiple misclassifications with the label BB.
  - In the 2D CNN model, the SS label was misclassified as label FF. Since the face fills a larger area when it is close to the camera, the SS and FF labels may contain more shared salient features.
  - Mean value for 2D CNN model was 70.7%, while the 3D CNN model was 91.07%.
3. Videos with a face positioned near the camera results: In all circumstances, the 3D CNN outperformed 2D CNN. This is due to the fact that the 3D CNN can manage temporal occlusions. The results are shown in Figure 8.
  - The NN label in the 2D CNN model has a very low recall. The NN label is misclassified mostly with the labels SS, BB, and LL. That happens because, inside one frame at a near camera distance, the face in all these three labels has almost the same shape.
  - FF had the lowest Recall value in the 2D CNN model. This comes from the deformations compared to a far-distance face position that may occur when the face is near the camera.
  - Figure 9 indicates the cause of SS’s low Recall value. The bottom of the image can be reached during face detection, and the image’s bottom will be transformed into a thick black line.
  - Mean value for 2D CNN model was 65.43%, while for the 3D CNN model, it was 88.08%.

Figure 9 shows some situations where 2D CNN may fail. For example, occasional occlusions can be hands on the face, extreme light brightness, and face near the camera, may lead the 2D CNN model to misclassification.

Overall, the 3D CNN had a better performance than 2D CNN:

1. 3D CNN can handle better temporal occlusions.
2. 2D CNN can handle light differences in the cases where the light is not occluding almost all of the face.
3. 3D CNN can address very strong light differences if some frames inside the image sequence are not occluding the full face of the driver.
4. 2D CNN cannot handle temporal occlusions well.
5. One interesting point for us was that the mean of the near and middle distance to the camera for the 3D CNN model was higher than the far distance. We believe that this is due to more forceful facial movements, which create a distinct pattern.

These characteristics make the 3D CNN model more robust than the 2D CNN model.

## 5. Contribution to Intelligent Transportation Systems

According to recent research [44], distraction is one of the primary causes of car accidents. If our vehicles feature an Advanced Driving Assistance Systems (ADAS) that identifies distractions ahead of time, the system will be able to not only warn but also take control of the situation in our near-autonomous vehicles, avoiding large-scale traffic accidents and contributing to traffic safety.

A driver’s gaze zone classifier inside an ADAS is crucial for detecting the driver’s situational awareness and readiness so that a partially or fully autonomous vehicle may take charge. Previous studies conducted their experiments in ideal conditions. Making this classification under uncontrolled settings is highly challenging, resulting in very low results in ongoing studies. The goal of our paper sequence is to create a high-performance driver gaze classifier under unconstrained situations. Having a high-performance system under unconstrained settings involves being able to recognize distractions with fewer errors, and so, substantially lowering traffic accidents.

## 6. Conclusions and Future Works

Current gaze classifiers suffer from a serious lack of implementations and assessments in unrestricted daily driving settings. Having a high-performance system under unconstrained conditions involves being able to recognize driver distractions with fewer errors and, therefore, dramatically lower traffic accidents.

To address this limitation in the current research, we are working through our different articles to identify and propose solutions to the misclassifications that may arise under various challenging situations in daily driving scenarios to create a highly robust driving monitoring system. In this paper, we presented a spatio-temporal analysis for handling misclassification during a gaze classification caused by the different face distances of the driver toward the cameras in a natural driving scenario by relying on features extracted from only selected frames of a video using convolutional descriptors for feature extraction.

To demonstrate our hypothesis, we made two models: one with 2D CNN and the other one with 3D CNN. Second, each model was evaluated during situations where different face positions to the camera were exposed. Third, we made a comparison between the models. All the experiments were conducted with our dataset since there is no publicly available dataset for this topic. The results showed that the 3D CNN had a better classification accuracy than a 2D CNN recognition model.

The implications of our findings are important because they can be a robust base for other gaze classifiers. Gaze classifiers can help make supportive systems for drivers or autonomous car systems for taking over control.

After the analysis of these results, future works should consider the results of this paper and integrate them into our current implementation of a robust driving monitoring system under unconstrained situations.

## Figures and Tables

**Table 1:** Labels included in our network. Each label represents a head direction towards one of the six standard driving gaze areas.

Label	Movement	Label	Movement
BB	Stare at the back mirror	FF	Stare to front
LL	Stare at the left window	RR	Stare at right window
NN	Stare at the navigator	SS	Stare at speed meter

## Data Availability

The data presented in this study are available on request from the corresponding author. The data is not publicly available because not all subjects consent to their faces being shown with the public.
